# Supplementary material for: Inflammation Status and Body Composition Predict Two-Year Mortality of Patients with Locally Advanced Head and Neck Squamous Cell Carcinoma under Provision of Recommended Energy Intake during Concurrent Chemoradiotherapy
Source: Biomedicines. 2022 Feb 6;10(2):388. doi: 10.3390/biomedicines10020388 (PMC8962429; doi:10.3390/biomedicines10020388)
Supplement: Supplementary file 1 [file biomedicines-10-00388-s001.zip › biomedicines-1527292-supplementary.pdf]

Supplementary Tables

Table S1. Correlation between pretreatment NIBs and body composition components using DXA before treatment in 123 patients with LAHNSCC undergoing CCRT

Pretreatment NIBs	Hb	WBC	PLT	TLC	Alb	CRP	NLR	PLR	PNI	LBM	TFM
Hb	---										
WBC	0.344	---									
PLT	0.585	<0.001*	---								
TLC	0.026*	<0.001*	0.704	---							
Alb	<0.001*	0.743	0.660	0.115	---						
CRP	0.141	0.089	0.125	0.329	0.336	---					
NLR	0.007*	0.001*	0.054	<0.001*	0.048*	0.311	---				
PLR	0.039*	0.014*	<0.001*	<0.001*	0.222	0.555	<0.001*	---			
PNI	0.001*	0.001*	0.385	<0.001*	<0.001*	0.698	<0.001*	<0.001*	---		
LBM	0.074	0.744	0.145	0.013*	0.003*	0.029*	0.055	0.011*	0.001*	---	
TFM	0.098	0.650	0.520	0.001*	0.088	0.599	0.035*	0.003*	0.001*	<0.001*	---

* $P < 0.05$, represents statistical significance.

Abbreviations: LAHNSCC, locally advanced head and neck squamous cell carcinoma; CCRT, concurrent chemoradiotherapy; NIBs, nutritional-inflammatory biomarkers; Hb, hemoglobin, g/dL; WBC, white blood cell, 10^3 cells/mm^3; PLT, platelet, 10^3/mm^3; TLC, total lymphocyte count, 10^3 cells/mm^3; Alb, albumin, g/dL; CRP, C-reactive protein, mg/dL; NLR, neutrophil-to-lymphocyte ratio; PLR, platelet-to-lymphocyte ratio; PNI, prognostic nutritional index; LBM, lean body mass, kg; TFM, total fat mass, kg; DXA, dual-energy X-ray absorptiometry.

Table S2. Association among OCC Factor 3, NOCC Factor 2, clinicopathological factors, treatment-related factors, nutritional/inflammatory markers, and body composition parameters assessed using DXA before CCRT in 123 patients with LAHNSCC

	OCC Factor 3		NOCC Factor 2	
	**Oral cavity with adjuvant CCRT**		**Non-oral cavity with primary CCRT**	
**Patient number**	**69**		**58**	
	**≤ 0.049 *vs.* > 0.049**		**≤ 0.3 *vs.* > 0.3**	
**Variables, expressed as number or mean ± standard deviation**				
**Patient number (n)**	**36 *vs.* 33**		**34 *vs.* 20**	
Clinicopathological factors		*P*-value*		*P*-value*
Age (years)	50.2 ± 7.8 *vs.* 56.9 ± 7.5	0.002*	54.7 ± 8.8 *vs.* 56.1 ± 8.1	0.623
Sex (male *vs.* female)	36 : 0 *vs.* 32 : 1	0.293	33: 1 *vs.* 18 : 2	0.274
TNM stage (III *vs.* IVA *vs.* IVB)	4 : 25 : 7 *vs.* 0 : 25 : 8	0.139	2: 21 : 11 *vs.* 3 : 14 :3	0.256
T status (T1-2 *vs.* T3-4)	4 : 32 *vs.* 4 : 29	0.896	9 : 25 *vs.* 11 : 9	0.049*
N status (N0-1 *vs.* N2-3)	17 : 19 *vs.* 13 : 20	0.512	8 : 26 *vs.* 3 : 17	0.452
Histological grade (well *vs.* MD *vs.* PD)	3 : 27 :6 *vs.* 5 : 24 : 4	0.623	2 : 19 :13 *vs.* 0 : 15 : 5	0.277
Smoking (no *vs.* yes)	3 : 33 *vs.* 3 : 30	0.910	1 : 33 *vs.* 4 : 16	0.052
Alcohol (no *vs.* yes)	10 : 26 *vs.* 8 :25	0.738	9 : 25 *vs.* 4 :16	0.591
Betel nut (no *vs.* yes)	10 : 26 *vs.* 6 : 27	0.345	17 : 17 *vs.* 10 : 10	1.000
ECOG performance status (0 : 1 : 2)	2 : 30 : 4 *vs.* 0 : 31 : 2	0.278	1 : 32 : 1 *vs.* 5 : 15 : 0	0.036*
HN-CCI (0 *vs.* ≥ 1)	17 : 19 *vs.* 12 : 21	0.254	15 : 19 *vs.* 7 : 13	0.510
Tracheostomy (no *vs.* yes)	11 : 25 *vs.* 12 : 21	0.609	29 : 5 *vs.* 19 : 1	0.049*
PG-SGA (A *vs.* B *vs.* C)	6 : 20 : 10 *vs.* 7 : 18 : 8	0.872	5 : 18 : 11 *vs.* 0 : 15 : 5	0.124
Anthropometric data and blood NIB data				
**Before CCRT**				
BMI (kg/m^2)	22.7 ± 4.2 *vs.* 22.8 ± 4.4	0.922	21.1 ± 3.7 *vs.* 25.5 ± 3.0	< 0.001*
BW (kg)	63.5 ± 12.0 *vs.* 63.9 ± 13.4	0.127	57.9 ± 11.4 *vs.* 69.5 ± 9.7	< 0.001*

Hb (g/dL)	11.9 ± 1.5 *vs.* 11.4 ± 1.3	0.102	11.2 ± 1.6 *vs.* 13.4 ± 1.9	0.031*
WBC (×10³ cells/mm³)	7.4 ± 2.6 *vs.* 7.1 ± 2.5	0.658	7.3 ± 3.3 *vs.* 6.8 ± 2.3	0.573
Platelet (×10³ /mm³)	308.2 ± 129.5 *vs.* 377.0 ± 160.9	0.064	248.5 ± 70.9 *vs.* 256.3 ± 80.9	0.718
TLC (×10³ cells/mm³)	1.7 ± 0.6 *vs.* 1.6 ± 0.6	0.632	1.5 ± 0.4 *vs.* 2.0 ± 0.6	0.046*
Albumin (g/dL)	3.6 ± 0.7 *vs.* 3.7 ± 0.4	0.478	3.6 ± 0.3 *vs.* 3.9 ± 0.5	0.022*
CRP (mg/dL)	8.5 ± 11.6 *vs.* 14.2 ± 16.8	0.120	21.5 ± 9.8 *vs.* 13.2 ± 10.1	0.549
NLR	4.2 ± 5.4 *vs.* 3.2 ± 1.9	0.361	3.2± 3.5 *vs.* 2.5 ± 1.1	0.463
PLR	202.1 ± 29.2 *vs.* 243.9 ± 28.6	0.312	152.9 ± 20.7 *vs.* 218.3 ± 10.9	0.022*
PNI	47.9 ± 6.3 *vs.* 45.6 ± 5.8	0.103	44.6 ± 5.5 *vs.* 48.5 ± 5.3	0.031*
**Treatment-interval change (%)**				
ΔBMI%**	-7.6 ± 0.8 *vs.* 0.34 ± 1.1	< 0.001*	-3.2 ± 7.3 *vs.* -9.2 ± 8.5	0.008*
ΔBW%**	-7.8 ± 0.8 *vs.* -0.2 ± 0.8	< 0.001*	-3.2 ± 7.2 *vs.* - 8.5 ± 8.6	0.020*
ΔHb%**	-8.5 ± 2.6 *vs.* -7.8 ± 2.4	0.884	-8.5 ± 2.5 *vs.* -18.5 ± 2.6	0.023*
ΔWBC%**	-17.7 ± 8.8 *vs.* -25.9 ± 5.2	0.436	-23.8 ± 8. *vs.* -24.2. ± 8.	0.97
ΔPlatelet%**	-14.4 ± 5.6 *vs.* -27.1 ± 4.5	0.079	-14.8 ± 5. *vs.* -12.5 ± 10.8	0.860
ΔTLC%**	-38.9 ± 9.8 *vs.* -39.4 ± 6.2	0.964	-56.1 ± 31 *vs.* -45.1 ± 39.3	0.276
ΔAlbumin%**	8.3 ± 5.0 *vs.* 4.9 ± 3.0	0.571	5.6 ± 3.7 *vs.* 0.2 ± 2.5	0.011*
ΔCRP%**	91.6 ± 11.5 *vs.* 75.6 ± 28.7	0.587	48.3 ± 12.8 *vs.* 47.7 ± 31.7	0.549
ΔNLR%**	2.6 ± 6.0 *vs.* 7.8 ± 21.1	0.287	7.4 ± 2.5 *vs.* 4.8 ± 1.6	0.441
ΔPLR%**	202.1 ± 29.3 *vs.* 223.9 ± 28.6	0.339	276.3 ± 99.6 *vs.* 220.5 ± 45.8	0.239
ΔPNI%**	-7.1 ± 2.3 *vs.* -2.7 ± 3.1	0.271	-11.2 ± 12.1 *vs.* -8.5 ± 14.1	0.457
**DXA-related measurements**				
**Before CCRT**				
LBM (kg)	43.9 ± 5.3 *vs.* 43.7 ± 5.1	0.889	42.7 ± 1.3 *vs.* 45.0± 1.9	0.025*
TFM (kg)	16.8 ± 8.0 *vs.* 17.4 ± 9.7	0.745	12.7 ± 7.4 *vs.* 21.6 ± 4.1	< 0.001*
ASM (kg)	18.7 ± 3.1 *vs.* 18.1 ± 3.0	0.472	17.6 ± 4.1 *vs.* 19.5 ± 2.3	0.031*

Android (%)	29.2 ± 13.0 *vs.* 30.2± 13.9	0.716	24.4 ± 8.4 *vs.* 40.0± 4.9	< 0.001*
Gynoid (%)	25.4 ± 7.7 *vs.* 26.0 ± 9.0	0.774	21.6 ± 4.3 *vs.* 30.5 ± 4.4	< 0.001*
**Treatment-interval change (%)**				
ΔLBM%**	-9.8 ± 2.0 *vs.* -2.0 ± 4.1	< 0.001*	-4.8 ± 7.2 *vs.* -6.8 ± 5.8	0.302
ΔTFM%**	-6.8 ± 3.0 *vs.* 2.0 ± 2.3	0.012*	-3.3 ± 23.7 *vs.* -10.2 ± 11.5	0.001*
ΔASM%**	-13.7 ± 3.6 *vs.* -2.5 ± 5.9	< 0.001*	-7.8 ± 10.9 *vs.* -8.5 ± 7.8	0.238
ΔAndroid%**	-1.6 ± 4.2 *vs.* -2.4 ± 2.8	0.438	0.9± 35.1 *vs.* -7.9 ± 12.4	0.213
ΔGynoid%**	1.1 ± 0.5 *vs.* 1.0 ± 0.4	0.939	4.83 ±27.2 *vs.* 0.6 ± 111.7	0.467
**Mean daily calorie intake during CCRT (kcal/kg/day)**	26.7 ± 7.8 *vs.* 30.7 ± 9.1	0.039*	29.5 ± 9.6 *vs.* 25.4 ± 4.0	0.022*
**< 30 : ≥ 30**	27 : 9 *vs.* 21 : 11	0.301	24 : 10 *vs.* 19 : 1	0.031*
**Mean daily protein intake during CCRT (g/kg/day, median)**	1.0 ± 0.8 *vs.* 1.1 ± 1.8	0.441	1.2 ± 1.1 *vs.* 0.8 ± 1.9	0.322
**Feeding tube placement (%)**	58.3 : 78.8	0.069	66.9 : 28.4	0.001*
**Mean days of feeding tube placement during CCRT (median)**	27.3 ± 4.8 *vs.* 42.4 ± 5.0	0.033*	26.0 ± 4.8 *vs.* 15.3 ± 5.2	0.011*
Treatment-associated factors				
**CCRT Regimen**				
RT dose (Gy)	64.1 ± 3.6 *vs.* 64.6 ± 4.1	0.571	69.8 ± 3.5 *vs.* 69.7 ± 2.6	0.862
RT fractions	31.8 ± 1.4 *vs.* 32.2 ± 1.8	0.256	33.6 ± 1.7 *vs.* 33.3 ± 0.9	0.463
RT duration (days)	47.8 ± 5.7 *vs.* 48.5 ± 4.0	0.660	52.3 ± 9.6 *vs.* 50.5 ± 4.3	0.417
Cisplatin dose (mg/m^2)	237.2 ± 15.3 *vs.* 239.8 ± 4.4	0.599	213.8± 10.8 *vs.* 212.3 ± 16.1	0.934
**CCRT-induced grade 3/4 toxicity**				
Dermatitis (no *vs.* yes)	35 : 1 *vs.* 31 : 2	0.504	33 : 1 *vs.* 19 : 1	0.699
Pharyngitis (no *vs.* yes)	34 : 2 *vs.* 31 : 2	0.719	26 : 8 *vs.* 18 : 2	0.216
Infection (no *vs.* yes)	34 : 2 *vs.* 27 : 6	0.405	22 : 12 *vs.* 15 : 5	0.432

Mucositis (no *vs.* yes)	26 : 10 *vs.* 25 : 8	0.507	25 : 9 *vs.* 15 : 5	0.909
Emesis (no *vs.* yes)	32 : 4 *vs.* 31 : 2	0.457	31 : 3 *vs.* 19 : 1	0.601
Anemia (no *vs.* yes)	33 : 3 *vs.* 31 : 2	0.716	30 : 4 *vs.* 17 : 3	0.733
Neutropenia (no *vs.* yes)	22 : 14 *vs.* 24 : 9	0.307	22 : 12 *vs.* 11 : 9	0.480
Thrombocytopenia (no *vs.* yes)	33 : 3 *vs.* 31 : 2	0.716	29 : 5 *vs.* 18 : 2	0.619
2-year mortality rate (%)	30.6 *vs.* 57.6	0.024*	41.2 *vs.* 10.5	0.015*

*The *P*-value was determined using the Chi-square test (for sex, TNM stage, T status, N status, histological grade, ECOG performance status, HN-CCI, PG-SGA smoking, alcohol, betel nut, tracheostomy, percentage of feeding tube placement, mean daily calorie during CCRT ≥ 30 kcal/kg/day, and all CCRT-induced grade 3/4 toxicities), independent *t*-test (age, RT dose, RT fraction, RT days, cisplatin dose, BMI, BW, Hb, WBC, platelet, TLC, albumin, CRP, NLR, PLR, PNI, and 2-year mortality rate), or Mann–Whitney tests (mean daily calorie intake, mean daily protein intake, mean days of feeding tube placement, all DXA-related parameters, and all treatment-interval changes in anthropometric data and blood NIB data). Statistical significance was set at $P < 0.05$.

**Δ indicates a value obtained by subtracting the pretreatment value from the post-treatment value. % indicates (Δ value/ the pretreatment value) ×100%

Abbreviations: LAHNSCC, locally advanced head and neck squamous cell carcinoma; OCC, oral cavity cancer; NOCC, non-oral cavity cancer; CCRT, concurrent chemoradiotherapy; SD, standard deviation; MD, moderately differentiated; PD, poorly differentiated; HN-CCI, Charlson comorbidity index; ECOG, Eastern Cooperative Oncology Group; PG-SGA, Patient-Generated Subjective Global Assessment; BW, body weight; BMI, body mass index; Hb, hemoglobin; WBC, white blood cell; TLC, total lymphocyte count; CRP, C-reactive protein; NLR, neutrophil-to-lymphocyte ratio; PLR, platelet-to-lymphocyte ratio; PNI, prognostic nutritional index; DXA, dual-energy X-ray absorptiometry; LBM, lean body mass; TFM, total fat mass; ASM, appendicular skeletal mass.

Table S3. Association among ECOG performance status, age, clinicopathological factors, treatment-related factors, nutritional/inflammatory markers, and body composition parameters before CCRT in 123 patients with LAHNSCC, as assessed using DXA

	**ECOG performance status**		**Age**	
	**Oral cavity cancer with adjuvant CCRT**		**Non-oral cavity cancer with primary CCRT**	
**Included patient number**	**69**		**54**	
	**< 2 *vs.* = 2**		**≤ 52.5 *vs.* > 52.5**	
**Variables, expressed as number or mean±standard deviation**				
**Patient number (n)**	**63 *vs.* 6**		**21 *vs.* 33**	
Clinicopathological factors		*P*-value*		*P*-value*
Age (years)	53.2 ± 1.0 *vs.* 53.8 ± 4.3	0857	47.8 ± 7.4 *vs.* 60.1 ± 5.0	< 0.001*
Sex (male *vs.* female)	62 : 1 *vs.* 6 : 0	0.847	20 : 1 *vs.* 31 : 2	0.839
TNM stage (III *vs.* IVA *vs.* IVB)	4 : 14 : 15 *vs.* 0 : 6 : 0	0.287	3 : 14 : 4 *vs.* 2 : 21 : 10	0.453
T status (T1-2 *vs.* T3-4)	8 : 55 *vs.* 0 : 6	0.353	6 : 15 *vs.* 14 : 19	0.304
N status (N0-1 *vs.* N2-3)	25 : 38 *vs.* 5 : 1	0.039*	4 : 17 *vs.* 7 : 26	0.826
Histological grade (well *vs.* MD *vs.* PD)	7 : 47 :9 *vs.* 1 : 4 : 1	0.899	0 : 13 :8 *vs.* 2 : 21 : 10	0.469
Smoking (no *vs.* yes)	5 : 58 *vs.* 1 : 5	0.468	3 : 18 *vs.* 2 : 31	0.609
Alcohol (no *vs.* yes)	16 : 47 *vs.* 2 :4	0.672	4 : 17 *vs.* 9 :24	0.924
Betel nut (no *vs.* yes)	14 : 49 *vs.* 2 : 4	0.538	9 : 12 *vs.* 18 : 15	0.402
ECOG performance status (0 : 1 : 2)	2 : 61 : 0 *vs.* 0 : 0 : 6	0.278	2 : 19 : 0 *vs.* 4 : 28 : 1	0.684
HN-CCI (0 *vs.* ≥ 1)	27 : 36 *vs.* 3 : 3	0.736	12 : 9 *vs.* 13 : 23	0.056
Tracheostomy (no *vs.* yes)	23 : 40 *vs.* 0 : 6	0.043*	16 : 5 *vs.* 28 : 5	0.425
PG-SGA (A *vs.* B *vs.* C)	12 : 34 : 17 *vs.* 1 : 4 : 1	0.819	1 : 15 : 5 *vs.* 4 : 18 : 11	0.419
Anthropometric data and blood NIB data				
**Before CCRT**				
BMI (kg/m²)	22.9 ± 4.4 *vs.* 20.9 ± 4.1	0.022*	23.3 ± 4.4 *vs.* 22.3 ± 3.8	0.406
BW (kg)	63.9 ± 3.4 *vs.* 60.1 ± 2.6	0.032*	66.0 ± 9.3 *vs.* 59.3 ± 5.8	0.033*

Hb (g/dL)	11.7 ± 1.4 *vs.* 11.7 ± 1.2	0.923	12.4 ± 1.4 *vs.* 11.7 ± 1.8	0.139
WBC (×10^3 cells/mm^3)	7.0 ± 2.3 *vs.* 9.6 ± 3.8	0.163	7.4 ± 3.8 *vs.* 6.9 ± 2.4	0.534
Platelet (×10^3 /mm^3)	330.1 ± 17.9 *vs.* 457.0 ± 71.6	0.044*	247.5 ± 69.5 *vs.* 253.5 ± 79.8	0.766
TLC (×10^3 cells/mm^3)	1.6 ± 0.6 *vs.* 1.8 ± 0.9	0.540	1.9 ± 0.7 *vs.* 1.7 ± 0.6	0.256
Albumin (g/dL)	3.8 ± 0.5 *vs.* 3.5 ± 0.4	0.028*	4.0 ± 0.6 *vs.* 3.6 ± 0.5	0.024*
CRP (mg/dL)	9.9 ± 13.3 *vs.* 24.8 ± 27.0	0.239	29.5 ± 70.4 *vs.* 15.3 ± 75.8	0.213
NLR	3.2 ± 6.6 *vs.* 20.9 ± 10.9	0.031*	2.4 ± 0.6 *vs.* 3.3 ± 0.5	0.022*
PLR	210.2 ± 19.4 *vs.* 347.2 ± 11.5	0.011*	141.2 ± 50.8 *vs.* 148.8 ± 1218.0	0.784
PNI	46.7 ± 6.1 *vs.* 47.8 ± 7.2	0.667	50.6 ± 4.1 *vs.* 46.8 ± 5.6	0.009*
**Treatment-interval change (%)**				
ΔBMI%**	-4.1 ± 6.6 *vs.* -2.2 ± 6.1	0.134	-4.9 ± 10.2 *vs.* -5.7 ± 6.8	0.737
ΔBW%**	-4.5 ± 6.8 *vs.* -4.0 ± 5.4	0.444	-5.0 ± 9.8 *vs.* –5.5 ± 6.4	0.830
ΔHb%**	-7.9 ± 1.8 *vs.* -10.7 ± 4.9	0.651	-11.7 ± 11.1 *vs.* -11.8 ± 15.5	0.966
ΔWBC%**	-17.7 ± 8.8 *vs.* -25.9 ± 5.2	0.436	-26.8 ± 33.9 *vs.* -22.2 ± 49.0	0.714
ΔPlatelet%**	-18.4 ± 30.4 *vs.* -44.7 ± 22.1	0.075	-10.5 ± 40.8 *vs.* -16.1 ± 38.0	0.618
ΔTLC%**	-37.2 ± 50.7 *vs.* -59.9 ± 19.2	0.281	-56.3 ± 34.2 *vs.* -49.5 ± 35.3	0.495
ΔAlbumin%**	5.1 ± 3.3 *vs.* 3.7 ± 5.0	0.491	6.4 ± 22.2 *vs.* 2.9 ± 15.9	0.632
ΔCRP%**	72.4 ± 41.5 *vs.* 83.1 ± 27.3	0.389	57.4 ± 34.1 *vs.* 66.0 ± 69.3	0.221
ΔNLR%**	3.2 ± 6.6 *vs.* 20.9 ± 43.2	0.287	9.2 ± 18.2 *vs.* 4.6 ± 6.5	0.280
ΔPLR%**	111.1 ± 25.7 *vs.* 99.4 ± 240.1	0.543	335.1 ± 71.8 *vs.* 194.2 ± 194.6	0.145
ΔPNI%**	-4.4 ± 16.1 *vs.* -8.7 ± 9.5	0.260	-9.7 ± 12.1 *vs.* -10.6 ± 13.5	0.787
**DXA-related measurements**				
**Before CCRT**				
LBM (kg)	44.2 ± 5.1 *vs.* 38.9 ± 2.8	0.015*	46.0 ± 7.1 *vs.* 42.0 ± 6.3	0.034*
TFM (kg)	16.8 ± 9.1 *vs.* 19.1 ± 6.0	0.125	17.0 ± 6.6 *vs.* 15.3 ± 5.7	0.231
ASM (kg)	18.8 ± 3.0 *vs.* 15.3 ± 1.7	0.007*	20.2 ± 3.9 *vs.* 17.7 ± 3.4	0.017*

Android (%)	28.8 ± 13.5 *vs.* 37.5± 9.5	0.131	30.8 ± 10.6 *vs.* 29.7± 10.5	0.721
Gynoid (%)	25.4 ± 8.2 *vs.* 27.7 ± 7.8	0.224	24.6 ± 6.4 *vs.* 25.1 ± 5.9	0.787
**Treatment-interval change (%)**				
ΔLBM%**	-6.2 ± 5.7 *vs.* -4.8 ± 5.0	0.599	-5.1 ± 7.2 *vs.* -5.9 ± 6.5	0.700
ΔTFM%**	-3.0 ± 14.7 *vs.* 1.8 ± 9.7	0.499	-2.1 ± 4.2 *vs.* -1.9 ± 2.6	0.238
ΔASM%**	-7.3 ± 7.2 *vs.* -13.9 ± 10.3	0.041*	-5.8 ± 9.1 *vs.* -9.4± 10.0	0.197
ΔAndroid%**	0.4 ± 22.3 *vs.* 0.5 ± 10.8	0.224	3.4 ± 7.9 *vs.* -2.9 ± 24.6	0.262
ΔGynoid%**	3.5 ± 11.1 *vs.* 13.0 ± 11.9	0.219	7.9 ± 3.0 *vs.* 1.1 ± 15.7	0.175
**Mean daily calorie intake during CCRT (kcal/kg/day)**	28.3 ± 8.5 *vs.* 31.9 ± 9.9	0.334	25.2 ± 8.1 *vs.* 25.8 ± 7.0	0.756
**< 30 : ≥ 30**	45 : 18 *vs.* 3 : 3	0.276	16 : 5 *vs.* 27 : 6	0.617
**Mean daily protein intake during CCRT (g/kg/day, median)**	1.1 ± 0.8 *vs.* 1.1 ± 1.8	0.742	1.1 ± 1.1 *vs.* 1.1 ± 1.2	0.802
**Feeding tube placement (no *vs.* yes)**	21 : 42 : 1 : 5	0.403	11 : 10 *vs.* 15 : 18	0.6193
**Mean days of feeding tube placement during CCRT (median)**	32.4 ± 3.6 *vs.* 56.5 ± 11.4	0.035*	18.7 ± 5.7 *vs.* 22.8 ± 4.8	0.473
Treatment-associated factors				
**CCRT Regimen**				
RT dose (Gy)	64.5 ± 2.8 *vs.* 62.1 ± 1.1	0.035*	69.7 ± 3.1 *vs.* 69.9 ± 3.2	0.806
RT fractions	32.0 ± 1.5 *vs.* 31.7 ± 2.7	0.608	33.6 ± 1.4 *vs.* 33.4 ± 1.5	0.618
RT duration (days)	48.5 ± 4.8 *vs.* 48.3 ± 5.5	0.875	51.3 ± 9.7 *vs.* 51.8 ± 7.0	0.832
Cisplatin dose (mg/m^2)	238.7 ± 19.4 *vs.* 236.7 ± 32.0	0.823	233.3 ± 10.1 *vs.* 200.5 ± 10.5	0.029*
**CCRT-induced grade 3/4 toxicity**				
Dermatitis (no *vs.* yes)	60 : 3 *vs.* 6 : 0	0.585	20 : 1 *vs.* 32 : 1	0.743
Pharyngitis (no *vs.* yes)	60 : 3 *vs.* 4 : 2	0.010*	17 : 4 *vs.* 27 : 6	0.936
Infection (no *vs.* yes)	55 : 8 *vs.* 4 : 2	0.170	17 : 4 *vs.* 20 : 13	0.113

Mucositis (no *vs.* yes)	49 : 14 *vs.* 3 : 3	0.131	18 : 3 *vs.* 22 : 11	0.119
Emesis (no *vs.* yes)	57 : 6 *vs.* 6 : 0	0.429	20 : 1 *vs.* 30 : 3	0.554
Anemia (no *vs.* yes)	58 : 5 *vs.* 6 : 0	0.474	18 : 3 *vs.* 29 : 4	0.813
Neutropenia (no *vs.* yes)	42 : 21 *vs.* 4 : 2	1.000	10 : 11 *vs.* 23 : 10	0.105
Thrombocytopenia (no *vs.* yes)	59 : 4 *vs.* 6 : 0	0.525	18 : 3 *vs.* 29 : 4	0.813
2-year mortality rate (%)	39.7 *vs.* 83.3	0.012*	14.3 *vs.* 39.4	0.042*

*The *P*-value was determined using the Chi-square test (for sex, TNM stage, T status, N status, histological grade, ECOG performance status, HN-CCI, PG-SGA smoking, alcohol, betel nut, tracheostomy, percentage of feeding tube placement, mean daily calorie intake during CCRT ≥ 30 kcal/kg/day **30** and all CCRT-induced grade 3/4 toxicities), independent *t*-test (age, RT dose, RT fraction, RT days, cisplatin dose, BMI, BW, Hb, WBC, platelet, TLC, albumin, CRP, NLR, PLR, PNI, and 2-year mortality rate), or Mann–Whitney tests (mean daily calorie intake, mean daily protein intake, mean days of feeding tube placement, all DXA-related parameters, and all treatment-interval changes in anthropometric data and blood NIB data). Statistical significance was set at $P < 0.05$.

**Δ indicates the value obtained by subtracting the pretreatment value from the post-treatment value. % indicates (Δ value/pretreatment value) × 100%

Abbreviations: LAHNSCC, locally advanced head and neck squamous cell carcinoma; OCC, oral cavity cancer; NOCC, non-oral cavity cancer; CCRT, concurrent chemoradiotherapy; SD, standard deviation; MD, moderately differentiated; PD, poorly differentiated; HN-CCI, Charlson comorbidity index; ECOG, Eastern Cooperative Oncology Group; PG-SGA, Patient-Generated Subjective Global Assessment; BW, body weight; BMI, body mass index; Hb, hemoglobin; WBC, white blood cell; TLC, total lymphocyte count; CRP, C-reactive protein; NLR, neutrophil-to-lymphocyte ratio; PLR, platelet-to-lymphocyte ratio; PNI, prognostic nutritional index; DXA, dual-energy X-ray absorptiometry; LBM, lean body mass; TFM, total fat mass; ASM, appendicular skeletal mass.

Table S4. Baseline characteristics of 123 patients with LAHNSCC in the complete CCRT subgroup and 42 incomplete CCRT/data collection subgroup undergoing CCRT stratified by tumor locations and CCRT settings

	OCC with adjuvant CCRT			NOCC with primary CCRT		
	CCRT completion			CCRT completion		
**Variables, expressed as Numbers (%) or mean±SD**	**Yes**	**No**	***P*-value**	**Yes**	**No**	***P*-value**
Patient number	69 (78.4)	19 (21.6)		54 (70.1)	23 (29.9)	
Clinicopathological characteristics						
Age (years)	53.2 ± 8.4	53.5 ± 7.8	0.873	55.3 ± 8.5	57.5 ± 8.3	0.295
Sex (male : female)	68 (98.6) : 1 (1.4)	19 (100.0) : 0 (0.0)	0.598	51 (94.4) : 3 (5.6)	22 (95.7) : 1 (4.3)	0.827
Tumor location (OCC/NOCC)			0.559			0.309
Buccal mucosa/Tonsil	20 (29.0)	5 (26.3)		13 (24.1)	2 (8.7)	
Tongue/Tongue base	28 (40.6)	5 (26.3)		6 (11.1)	4 (17.4)	
Gingiva/Soft palate	13 (18.9)	4 (21.1)		3 (5.6)	2 (8.7)	
Mouth floor/Hypopharynx	3 (4.3)	2 (10.4)		24 (44.4)	9 (39.1)	
Retromolar/Larynx	2 (2.9)	1 (5.3)		8 (14.8)	6 (26.1)	
Lip	2 (2.9)	1 (5.3)		---	---	
Hard palate	1 (1.4)	1 (5.3)		--	--	
TNM stage (III *vs.* IVA *vs.* IVB)	4(5.8):50(72.5):15(21.7)	1(5.3):12(63.2):6(31.5)	0.672	5(9.3):35(64.8):14(25.9)	6(26.1):12(52.2):5(201.7)	0.155
T status (T0-2 *vs.* T3-4)	8 (11.6) : 61(88.4)	4 (21.1) : 15 (78.9)	0.287	20 (37.0) : 34 (63.0)	7 (30.4) : 16 (69.6)	0.578
N status (N0-1 *vs.* N2-3)	30 (43.5) : 39 (56.5)	11 (57.9) : 8 (42.1)	0.265	11 (20.4) : 43 (79.6)	6 (26.1) : 17 (73.9)	0.58
ECOG performance status (0 : 1 : 2)	2(2.9):61(86.4):6(8.6)	1(5.3):16(84.2):2(10.5)	0.552	6(11.1):47(87.0):1(1.9)	2(8.7):19(82.6):2(8.7)	0.413
Histological grade (1 : 2 : 3)	8(11.6):51(73.9):10(14.5)	3(15.8):14(73.7):2(10.5)	0.826	2(3.7):34(63.0):18(33.3)	1 (4.3):15(65.2):7(30.4)	0.967
Smoking (no : yes)	6 (8.7) : 63 (91.3)	3 (15.8) : 16 (84.2)	0.366	5 (9.3) : 49 (90.7)	0 (0.0) : 23 (100.0)	0.131
Alcohol (no : yes)	18 (26.1) : 51 (73.9)	7 (36.8) : 12 (63.2)	0.357	13 (24.1) : 41 (75.9)	4 (17.4) : 19 (82.6)	0.518
Betel nut (no : yes)	16 (23.2) : 53 (76.8)	6 (31.6) : 13 (68.4)	0.455	27 (50.0) : 27 (50.0)	12 (52.2) : 11 (47.8)	0.861
HN-CCI (0 *vs.* 1 *vs.* 2 *vs.* ≥3)	29(42.1):15(21.7):6(8.7):19(27.5)	13(68.4):3(15.8):3(15.8):0(0.0)	0.115	22(40.7):17(31.5):6(11.1):9(16.7)	15(65.2):4(17.4):4(17.4):0(0.0)	0.075
Tracheostomy (no : yes)	23 (33.3) : 46 (66.7)	10 (52.6) : 9 (47.4)	0.124	44 (81.5) : 10 (18.5)	17 (73.9) : 6 (26.1)	0.454

Nutritional and inflammatory markers before CCRT						
BW (kg)	63.6 ± 12.6	61.4 ± 10.1	0.026*	62.1 ± 12.1	66.8 ± 12.9	0.199
BMI (kg/m²)	22.7 ± 4.3	22.6 ± 3.6	0.525	22.7 ± 4.0	24.8 ± 5.5	0.113
Hb (g/dL)	11.7 ± 1.5	12.1 ± 2.6	0.400	11.9 ± 1.7	11.2 ± 2.2	0.150
WBC (×10³ cells/mm³)	7.3 ± 2.5	8.7 ± 2.8	0.062	7.1 ± 2.9	7.4 ± 2.8	0.716
Platelet count (×10³/mm³)	341.1 ± 148.4	274.5 ± 187.1	0.066	251.4 ± 75.3	265.5 ± 86.8	0.473
TLC (×10³ cells/mm³)	1.6 ± 0.6	1.8 ± 0.5	0.240	1.8 ± 0.7	1.9 ± 0.6	0.283
Albumin (g/dL)	3.8 ± 0.6	3.9 ± 0.7	0.488	3.8 ± 0.5	3.9 ± 0.5	0.427
CRP (mg/dL)	11.2 ± 1.8	7.9 ± 7.2	0.431	18.7 ± 6.6	8.7 ± 15.3	0.398
PG-SGA (well vs. moderate vs. severe) before CCRT	13(18.8):38(55.1):18(26.1)	12(63.2):6(31.6):1(5.3)	0.001*	5 (9.3):33(61.1):16(29.6)	18 (78.3): 5 (21.7): 0(0.0)	< 0.001*
Body composition parameters before CCRT						
LBM (kg)	43.8 ± 5.1	44.4 ± 8.6	0.662	43.6 ± 6.8	45.6 ± 6.8	0.249
TFM (kg)	17.0 ± 8.8	16.4 ± 6.5	0.769	15.9 ± 6.0	15.4 ± 8.1	0.754
ASM (kg)	18.4 ± 3.0	18.9 ± 2.7	0.535	18.7 ± 3.6	19.2 ± 3.9	0.603

*indicates a significant *P*-value < 0.05. The analytical methods corresponding to data shown in Table S2 are identical to those in Table 3

Abbreviations: LAHNSCC, locally advanced head and neck squamous cell carcinoma; CCRT, concurrent chemoradiotherapy; OCC, oral cavity cancer; NOCC, non-oral cavity cancer; TNM, tumor node metastasis; ECOG, Eastern Collaboration Oncology Group; HN-CCI, head and neck Charlson Comorbidity Index; RT, radiotherapy; PG-SGA, Patient-Generated Subjective Global Assessment; BMI, body mass index; BW, body weight; Hb, hemoglobin; WBC, white blood cell count; TLC, total lymphocyte count; CRP, C-reactive protein; LBM, lean body mass; TFM, total fat mass; ASM, appendicular skeletal muscle mass; BMC, bone mineral content.

Independent *t*-tests were used for age, BW, BMI, Hb, TLC, and albumin levels. The Mann–Whitney test was used for WBC, platelet count, CRP, and all body composition parameters. The chi-square test was used for all categorical data.
